# Innovations in Egg Alternatives: Sustainable Sources, Novel Technologies, Challenges, and Future Perspectives

**DOI:** 10.3390/foods15203638

**Published:** 2026-10-13

**Authors:** Shahriyar Sahraeian, Behrokh Abdollahi, Jen-Yi Huang, Marco Garcia-Vaquero, Armin Mirzapour-Kouhdasht

**Affiliations:** 1 Department of Food Science and Technology, College of Agriculture, Shiraz University, Shiraz 7194684334, Iran; s40030168@hafez.shirazu.ac.ir or shahriyarsahraeian@gmail.com; 2 Department of Food Science and Technology, Gorgan University of Agricultural Sciences and Natural Resources, Gorgan 4913815739, Iran; behrokh.abdollahi_s99@gau.ac.ir; 3 Department of Food Science and Technology, The Ohio State University, Columbus, OH 43210, USA; huang.6338@osu.edu; 4 Department of Food, Agricultural, and Biological Engineering, The Ohio State University, Columbus, OH 43210, USA; 5 School of Agriculture and Food Science, University College Dublin, Belfield, D04 V1W8 Dublin, Ireland; marco.garciavaquero@ucd.ie; 6 Department of Nutrition, Dietetics & Food Sciences, Utah State University, Logan, UT 84322, USA

**Keywords:** egg alternatives, alternative proteins, emulsifier, food processing, health, alternative egg

## Abstract

Researchers are developing egg alternatives to match the nutritional, sensory, and techno-functional properties of conventional eggs, but no single alternative ingredient currently replicates the full multifunctionality of eggs. This review critically evaluates recent advances in egg-alternative systems, with an emphasis on alternative proteins and functional ingredients. Comparative evidence indicates that several legume proteins, like adzuki and cowpea, can provide strong egg-like functionality. Protein concentrates can also provide improved functional performance at lower concentrations than corresponding isolates, whereas cereal proteins generally face limitations related to solubility, foaming, and emulsification. Hydrocolloids, starches, fibers, and aquafaba can further improve gelation, emulsion stability, and foam structure, although their effects depend strongly on formulation and processing conditions. Despite these advances, the principal functional gap remains the simultaneous replication of egg functionalities within a single formulation. Nutritionally, plant-based systems commonly remain limited by incomplete amino acid profiles and lower levels or bioavailability of micronutrients such as vitamins. The evidence therefore supports a design strategy based on complementary protein blends, controlled protein–polysaccharide–lipid interactions, targeted fortification with attention to bioavailability, and processing technologies that improve functionality without compromising sensory quality. Future development should prioritize multifunctional formulations validated against egg benchmarks using standardized functional, nutritional, sensory, and digestibility measurements.

## 1. Introduction

Eggs are one of the most widely consumed foods, either as food or as a food ingredient in both household and industrial environments, due to their exceptional nutritional properties, providing high-quality proteins, vitamins, and minerals, along with excellent techno-functional attributes, such as emulsification, gelling, foaming, and binding properties [1,2]. These properties make eggs an essential ingredient for food formulation, and they are regularly incorporated into baked goods, sauces, and other processed foods. Moreover, eggs are considered staples in many parts of the world because they are affordable and provide high-quality nutrients for balanced diets [2].

Recent data show that the conventional egg market is very large and growing. Their established role in household consumption and food manufacturing, together with increasing interest in animal-free and alternative protein products, has stimulated the development of commercial egg-alternative systems. The global egg-alternative market has expanded substantially in recent years, with products based on plant proteins, starches, hydrocolloids, algae-derived ingredients, and other emerging technologies entering applications such as mayonnaise, bakery products, and scrambled-egg analogues [3,4]. This commercial development demonstrates the practical importance of egg alternatives; however, the continuing difficulty of reproducing the integrated nutritional, interfacial, rheological, thermal, and sensory properties of conventional eggs remains a fundamental scientific challenge.

The growing reliance on animal-derived products has prompted increased scrutiny. A transformation is underway in global food systems, as consumers, policymakers, and industries become increasingly aware of the sustainability and ethical aspects of animal-derived products [5]. This growing consumer demand is driven by a number of reasons, such as environmental concerns, health consequences of consuming these products, and animal welfare [6]. The conventional production systems of animal-derived products have been linked to negative environmental impacts, including greenhouse gas emissions, deforestation, and water pollution [5]. Specifically, approximately 15% of greenhouse gas emissions worldwide have been attributed to livestock farming [7]. Although eggs generally exhibit a lower environmental footprint than ruminant-derived products, their production still requires substantial feed, land, and water resources and contributes to greenhouse gas emissions through feed cultivation, transportation, and manure management. For example, life-cycle assessments indicate that production of 1 kg of eggs generates about 2–3 kg of CO_2_-eq in greenhouse gases, typically requires 5–7 m^2^ of cropland, and consumes 3000–3300 L of water [8]. In addition, intensive egg production systems have raised concerns regarding laying-hen welfare, including confinement practices, beak trimming, and management of male chicks, which have attracted increasing public and regulatory attention [9]. Egg production is also vulnerable to supply-chain disruptions caused by disease outbreaks such as highly pathogenic avian influenza, which can result in substantial economic losses, reduced egg availability, and price volatility [10,11]. Furthermore, eggs are among the most common food allergens, particularly in children, creating an additional demand for egg-free alternatives suitable for sensitive consumer groups [12]. Therefore, these environmental, ethical, economic, and health-related challenges have accelerated research and commercial efforts to develop sustainable and functional egg alternatives.

Regarding the environmental challenges, there is a need to promote new farming and food production practices that minimize the environmental impacts of food production while maintaining nutritional and safety attributes for the development of sustainable food systems that can feed the growing world population. Notably, sustainability frameworks generally call for a reduced consumption of animal-sourced foods rather than complete elimination. For example, recent dietary guidelines propose roughly a 1:1 ratio of animal to plant protein to achieve large emission cuts without full abstinence [13]. Likewise, experts emphasize that improving animal health and overall farming efficiency can dramatically cut livestock emissions while maintaining production levels.

Recent market trends have shown increased consumer preference for alternative foods and alternative protein sources [14,15]. This rapid change in consumer preferences has been propelled by the growing consciousness of the health benefits of alternative diets, which include diminishing risks of chronic diseases—including heart disease, diabetes, or even obesity [16,17]—as well as ethical concerns related to animals, with an increasing number of consumers seeking cruelty-free food alternatives [18]. The food sector has answered these demands with a wide array of innovative solutions, from alternative meats, fish, drinks and egg substitutes to laboratory-grown proteins [19,20,21]. For example, advanced manufacturing techniques such as extrusion and 3D food printing are now being applied to alternative ingredients to create meat- and fish-like products, and food technologists are increasingly upcycling agricultural byproducts, like brewer’s spent grains and oilseed meals, into protein-rich ingredient flours [11,22]. In parallel, cellular agriculture and fermentation approaches are enabling animal proteins to be produced in vitro. For instance, engineered microbes, such as yeasts, bacteria, or fungi, are employed to ferment feedstocks into animal-equivalent proteins, such as caseins, egg-white proteins, collagen, heme, etc., effectively “cultivating” traditional animal proteins without animals [23].

Egg alternatives stand out as one of the major innovation fronts in the food sector. The global vegan egg market is highly promising, valued at US$1.5 billion in 2021 and projected to expand at a compound annual growth rate (CAGR) of 8.3% through 2031 [3]. These products are produced to mimic the functional, nutritional, and sensory properties of eggs by using plant-based or other non-animal ingredients. Common types of ingredients encompass legumes (e.g., pea protein), cereals (e.g., rice protein), and hydrocolloids (e.g., xanthan gum) meant to mimic egg techno-functional attributes, such as emulsification, foaming, and binding [4].

However, these new egg alternatives must address multiple aspects to be considered suitable egg replacers. Their functionality must be considered, as eggs are a remarkably versatile ingredient, providing the structure, texture, and stability required by multiple food formulations [24,25]. Alternative eggs should ideally mimic these properties to gain consumer acceptance. The central scientific challenge in developing egg alternative systems is not the reproduction of a single property, but the simultaneous replication of the interconnected structural, interfacial, rheological, and nutritional functions of eggs. Egg white and egg yolk are compositionally and structurally distinct systems that provide complementary functionalities. For egg-white functionality, foaming depends on the ability of proteins to diffuse to the air–water interface, adsorb, partially unfold, and form a cohesive and viscoelastic interfacial layer capable of stabilizing incorporated air bubbles [26,27]. Upon heating, protein denaturation and subsequent intermolecular interactions generate a three-dimensional gel network that determines the firmness, water retention, and structural integrity of the final product. Plant proteins can reproduce individual aspects of these mechanisms, but differences in protein composition, molecular structure, surface hydrophobicity, sulfhydryl content, solubility, and unfolding/aggregation behavior can result in different interfacial and gelation responses [4,6]. Consequently, a protein with high foaming capacity does not necessarily provide an egg-like thermal gel, while a protein capable of forming strong gels may not provide adequate foam formation or interfacial stability.

The molecular complexity of egg yolk presents an additional challenge. Egg yolk contains phospholipids, lipoproteins, soluble proteins, and organized protein–lipid aggregates that collectively determine its emulsifying, rheological, optical, and sensory properties [28,29]. Replication therefore requires coordinated control of oil–water interfacial adsorption, droplet size and stability, continuous-phase rheology, and protein–lipid interactions. Plant proteins, starches, hydrocolloids, and plant oils can reproduce individual functions, but their molecular interactions and thermal responses differ from those of the native yolk matrix [4,6]. This helps explain why formulation strategies increasingly rely on combinations of proteins, polysaccharides, lipids, emulsifiers, and colorants rather than a single replacement ingredient. The nutritional attributes of egg alternatives are equally important, as eggs constitute a good source of high-quality protein, containing all essential amino acids along with important micronutrients like vitamin B12 and choline [1,30]. However, it is reported that high consumption of eggs may result in cardiovascular disease because of high cholesterol concentrations in egg yolk [3]. From a formulation perspective, replacing egg yolk can provide an opportunity to modify the nutritional composition of a food product by reducing or eliminating dietary cholesterol and incorporating plant-derived ingredients containing dietary fiber, phytosterols, and other bioactive compounds [3]. However, these potential nutritional advantages depend on the composition of the alternative formulation and should not be interpreted as evidence that egg alternatives are intrinsically healthier than conventional eggs. In addition, the nutritional consequences of substitution should be evaluated in terms of the complete formulation, including protein quality, lipid composition, micronutrient density, and bioavailability.

Currently, plant ingredient applications cannot replicate the nutritional profile of eggs from a single protein source; therefore, blends from multiple protein sources are regularly used to achieve a balanced amino acid profile [6]. On the other hand, another major hurdle for the widespread use of egg alternatives is their price, as currently available egg alternatives are significantly more costly when compared to eggs [31]. With further development in ingredient sourcing, processing technologies, and the scale-up of production, the price of egg alternatives is likely to decrease in the near future; however, it remains a major obstacle to the adoption of egg alternatives in larger markets.

Several recent reviews have addressed the development of vegan eggs and plant-based egg analogues, including their formulation, nutritional characteristics, techno-functional properties, and applications [3,32,33]. The present review is intended to complement these contributions by adopting a broader source-to-application perspective and by integrating recent developments reported by 2026. In particular, the review distinguishes itself by (i) systematically considering conventional and emerging protein sources, including legumes, cereals and pseudocereals, algae, insects and larvae, and fungi, while identifying cases in which evidence for direct egg-alternative applications remains limited; (ii) linking ingredient functionality and processing strategies to the specific structural and techno-functional requirements of egg white, egg yolk, and whole-egg replacement; (iii) emphasizing quantitative benchmarking against conventional eggs through measurable functional, rheological, thermal, water-holding, textural, nutritional, and sensory attributes; and (iv) integrating technological, nutritional, economic, sustainability, and commercialization constraints to identify gaps that remain between ingredient-level functionality and practical egg replacement. Accordingly, rather than providing another general inventory of egg-replacement ingredients, this review seeks to organize the available evidence around the multifunctionality of eggs and the requirements for designing more effective and application-specific alternative systems.

## 2. Eggs

### 2.1. Overview

Eggs are very versatile and widely used animal-derived foodstuffs, valued worldwide due to their unique sensory, functional, and nutritional properties [30,34]. Most commercialized eggs are from chickens, although a relatively small market for eggs from ducks, quails, and geese also exists [35]. Their broad global use is associated with their low cost, suitability for diverse dietary patterns, and their use as food ingredients incorporated into other food formulations to provide texture, flavor, and nutrition [30].

### 2.2. Chemical Composition and Nutritional Properties

**Figure 1:**
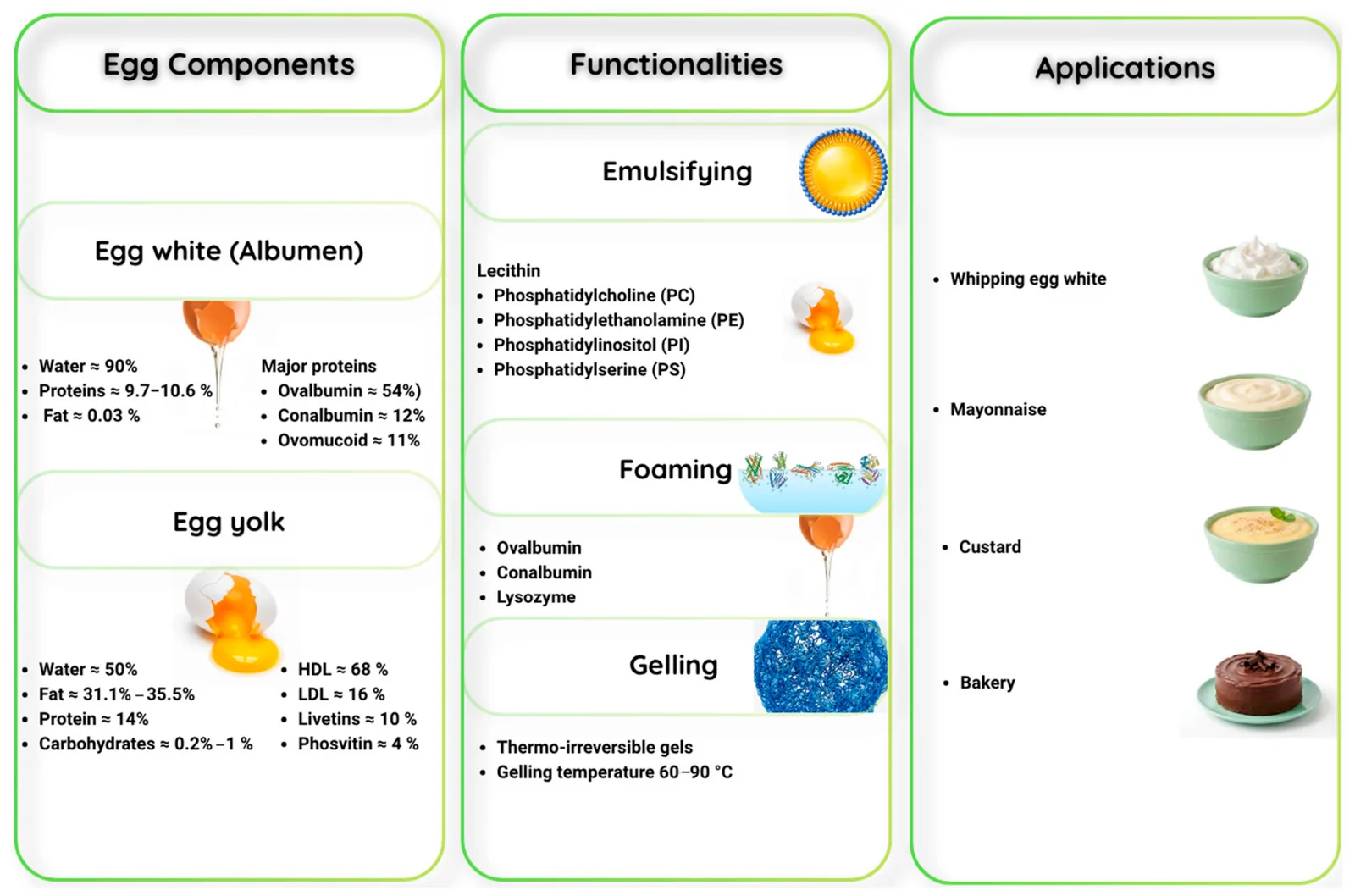
Schematic representation of egg components, including egg white (albumen) and egg yolk, and its functionalities with applications.

The chemical structure of eggs is quite intricate, encompassing water, proteins, lipids, carbohydrates, vitamins, and minerals. The egg white, or albumen and the egg yolk (Figure 1) are plainly the two main parts of an egg, and they clearly differ from each other chemically [36]. Water forms most of the edible part of egg white [36]. Egg white is also mainly composed of proteins (9.7–10.6%) while its fat content is negligible (0.03%) [37]. Table 1 summarizes the protein and amino acid profiles of egg white. As shown, ovalbumin is the predominant protein fraction in egg white, comprising over half of the total protein content (54.0%), followed by conalbumin (12.0%) and ovomucoid (11.0%). Furthermore, the amino acid profile underscores the high biological value of egg white, which shows a comprehensive array of essential amino acids, most notably leucine (0.95 g/100 g) and valine (0.84 g/100 g), alongside high concentrations of glutamic acid (1.52 g/100 g) and aspartic acid (0.85 g/100 g). Replicating this complex, highly bioavailable protein matrix remains a key nutritional benchmark and formulation challenge in developing viable alternative or synthetic egg products.

Egg yolk, an oil-in-water emulsion, is made up of lipids, proteins, carbohydrates, vitamins and minerals, among which lipids are the main compounds present, with fat content ranging from 31.1% to 35.5%, and a low amount of carbohydrates ranging between 0.2% and 1% [37]. The constituents are mainly high-density lipoprotein (HDL; 68%), low-density lipoprotein (LDL, 16%), livetins (10%) and phosvitin (4%) [24]. Egg yolk is a multi-structured system, containing insoluble protein aggregates known as granules (~50% of the proteins in egg yolk) and composed of HDLs (70%) and phosvitin (16%) that are covalently linked by phosphocalcic bridges. These protein aggregates are suspended in plasma, a clear yellow fluid rich in carotenoids and composed of 85% of LDL and 15% of livetins [38].

Eggs are considered the most nutritionally complete food because they offer a better balance of nutrients than any other food [34]. Eggs supply high-quality protein, including all nine essential amino acids, and are often used as a reference standard to measure the quality of proteins [39,40]. Eggs also contain important lipids, primarily located in the yolk, such as monounsaturated, polyunsaturated, and omega-3 fatty acids, which support normal brain function, hormone production, and cellular processes [41,42]. In addition, eggs provide essential vitamins and minerals, including vitamins A, D, E, K, and the B-complex, iron, zinc, phosphorus, and selenium, that are necessary for vision, bone health, metabolism, immune function, and antioxidant functions [30]. Furthermore, eggs are also a natural source of bioactive compounds, like lutein, zeaxanthin, and choline, associated with eye health and brain growth [43,44,45,46]. Overall, eggs contain a comprehensive range of nutrients and therefore form an important part of diets and are often used as a standard for comparison with alternative products.

### 2.3. Techno-Functional Properties

One of the main and well-recognized functions of eggs is their emulsifying capacity, primarily due to the lecithin in the yolk [28]. Lecithin (Figure 1) is a complex glycerophospholipid mixture that consists of phosphatidylcholine, phosphatidylethanolamine, phosphatidylinositol, and phosphatidylserine [47]. Lecithin is a natural surfactant that stabilizes oil-in-water emulsions such as those in mayonnaise, salad dressings and sauces [29]. Another important role of eggs is foaming, which is essentially a property of egg white. Whipping egg white can entrap air, producing stable foams and the light porous texture required for meringues, soufflés and angel food cakes [48]. The excellent foaming properties of eggs are related to ovalbumin, conalbumin and lysozyme, which unfold and form a network around air bubbles during the whipping process [49].

Another crucial techno-functional property of eggs of interest to the food industry includes gelling properties and color, amongst others. Upon heating, egg proteins denature and transform into a gel network that provides structure and texture to foods like custards, quiches and baked products [50,51]. These gelling properties are useful when eggs are used as a binding agent in products, such as meatballs [52] and patties [53]. Eggs are normally turned into thermo-irreversible gels at 60–90 °C, which is near their proteins’ denaturation temperature [32]. Such quantitative reference can be used by food scientists when designing food products containing egg alternatives. Reports show that plant proteins like soy, lentil, and chickpea possess higher denaturation temperatures ranging from 90 °C to 120 °C, while others like potato and RuBisCO have similar or lower denaturation temperatures [32]. Eggs also help retain moisture in baked goods, such as cakes and muffins [46]. In addition, egg yolk adds color and taste to food formulations [54], which is highly valued for its aesthetic appeal in pasta [26] and sauces [27].

For egg white alternatives, the principal benchmarks should include protein concentration, solubility, foaming capacity or overrun, foam stability, apparent viscosity, thermal transition behavior, gel strength, water-holding capacity, and texture after heating. Thus, successful alternatives should reproduce not only the final firmness of the heated product but also an appropriate sequence of unfolding, aggregation, and network formation. For egg yolk alternatives, quantitative evaluation should additionally consider emulsifying activity and stability, droplet-size distribution, apparent viscosity, shear-thinning behavior, interfacial stability, gel strength where applicable, and color. The complex plasma–granule organization of yolk means that successful replacement requires more than reproducing total lipid or protein content; the interfacial and rheological behavior of the resulting lipid–protein system must also be considered.

Accordingly, future egg-alternative studies should report the performance of alternative formulations relative to a conventional egg control using clearly defined analytical conditions. Studies should also provide quantitative values for overrun and foam stability, emulsifying indices, viscosity or flow parameters, thermal transition temperatures, gel strength, and texture. This benchmark-oriented approach would improve comparability among studies and facilitate the rational design of multifunctional egg-alternative systems.

Eggs have a wide range of applications in the production of baked goods, confections, and processed foods. In terms of structure and texture, egg white creates stable foams that are vital to the production of meringues, macarons, marshmallows, and nougats [55,56,57], while egg yolk contributes lipids to improve the flavor, tenderness, and mouthfeel of batters, custards, and ice cream [50,58,59]. In addition, eggs add leavening capacity by aerating batters [60], bind ingredients, retain moisture [61], and improve aesthetics by providing a surface glaze on baked products [62]. In a broader context of food processing, eggs serve as emulsifying agents in sauces, binding agents in meat matrices [52], and agents that improve noodle dough elasticity through textural manipulation [63]. Furthermore, the high biological value of eggs also lies in the use of their proteins in dietary supplements and functional foods that support recovery after physical exertion and promote well-being [64,65]. Given the diverse culinary and industry applications, developing alternatives to eggs presents significant challenges.

**Table 1:** Proteins and amino acid profile of dried egg white in fresh egg retrieved from Matsuoka and Sugano [66], Li et al. [67], and Belitz [68].

**Proteins**	**Contents (% of total protein)**
Ovalbumin	54.0
Conalbumin (Ovotransferrin)	12–13
Ovomucoid	11
Ovomucin	1.5–3.5
Lysozyme (Ovoglobulin G1)	3.4–3.5
Ovoglobulin G2	4.0
Ovoglobulin G3	4.0
Flavoprotein	0.8
Ovoglycoprotein	1.0
Ovomacroglobulin	0.5
Ovomacroglobulin (ovostatin)	0.5
Avidin	0.05
Cystatin (Ficin inhibitor)	0.05
**Amino acids**	**Contents (g/100 g edible portion)**
Alanine (Ala)	5.3
Arginine (Arg)	5.0
Aspartic Acid (Asx)	9.3
Cysteine (Cys)	2.5
Glutamic Acid (Glx)	12.0
Glycine (Gly)	3.2
Histidine (His) *	2.1
Isoleucine (Ile) *	4.4
Leucine (Leu) *	7.3
Lysine (Lys) *	6.1
Methionine (Met) *	3.2
Phenylalanine (Phe) *	5.1
Proline (Pro)	3.3
Serine (Ser)	6.0
Threonine (Thr) *	4.0
Tryptophan (Trp) *	1.3
Tyrosine (Tyr)	3.9
Valine (Val) *	5.8

* This symbol denotes essential amino acids within the table.

## 3. Egg Alternatives

### 3.1. Overview

Egg alternatives are made of alternative ingredients, and ideally, they aim to have equal or superior performance to that of eggs in terms of their functional, sensory, and nutritional properties. The terminology used for egg alternatives encompasses several distinct technological categories that should not be considered synonymous. In this review, egg alternative is used as an umbrella term referring to food systems designed to replace eggs, egg white, or egg yolk in terms of one or more functional, sensory, nutritional, or processing attributes. Within this broader category, plant-based egg alternatives specifically refer to formulations in which the principal functional ingredients are derived from terrestrial plants, including legumes, cereals, oilseeds, and other plant sources. Hydrocolloids, starches, fibers, and plant-derived emulsifiers may be incorporated as complementary ingredients to provide or enhance egg-like functionality. Non-plant biomass-derived alternatives include proteins or functional ingredients obtained from algae, fungi, and other microbial or biomass sources. These materials are distinguished from conventional plant proteins based on their biological origin, although they may be incorporated into formulations intended to provide plant-forward or animal-free alternatives. Their inclusion in this review therefore reflects their potential as alternative protein or functional ingredients rather than their classification as plant-derived ingredients. A third category is represented by fermentation-derived egg proteins, particularly proteins produced through precision fermentation using engineered microorganisms. In contrast to plant-based alternatives, these systems may produce proteins that are molecularly identical or functionally analogous to specific egg proteins without requiring conventional egg production. Insect- and insect-larvae-derived ingredients have also been discussed in this study as they can serve as potential sources of animal-free ingredients.

Egg alternatives are developed to align with consumer interest in household and industrial sustainable, allergen-free, and animal-free options. They can be offered as whole product, egg white, and egg yolk alternatives, aiming to mimic each part of the egg based on the needs of the food industry [69,70]. Thereby, whole egg alternatives seek to perform similarly to whole eggs in applications like scrambled eggs, omelets, quiche, and baking [71]. Egg white or egg yolk alternatives are designed as high-protein and lipid-rich systems, respectively, for specific applications in other products, including meringues, mousses, bakery products, and confectionery products [72]. Alternative egg white and egg yolk alternatives can also be found in liquid or powder forms. Liquid formats are most commonly used in scrambled egg and omelet applications, while powdered forms are primarily used for baking and dry mix formulations, as they have longer shelf stability and are easier to store compared to their liquid counterparts [72].

### 3.2. Formulation of Egg Alternatives

**Figure 2:**
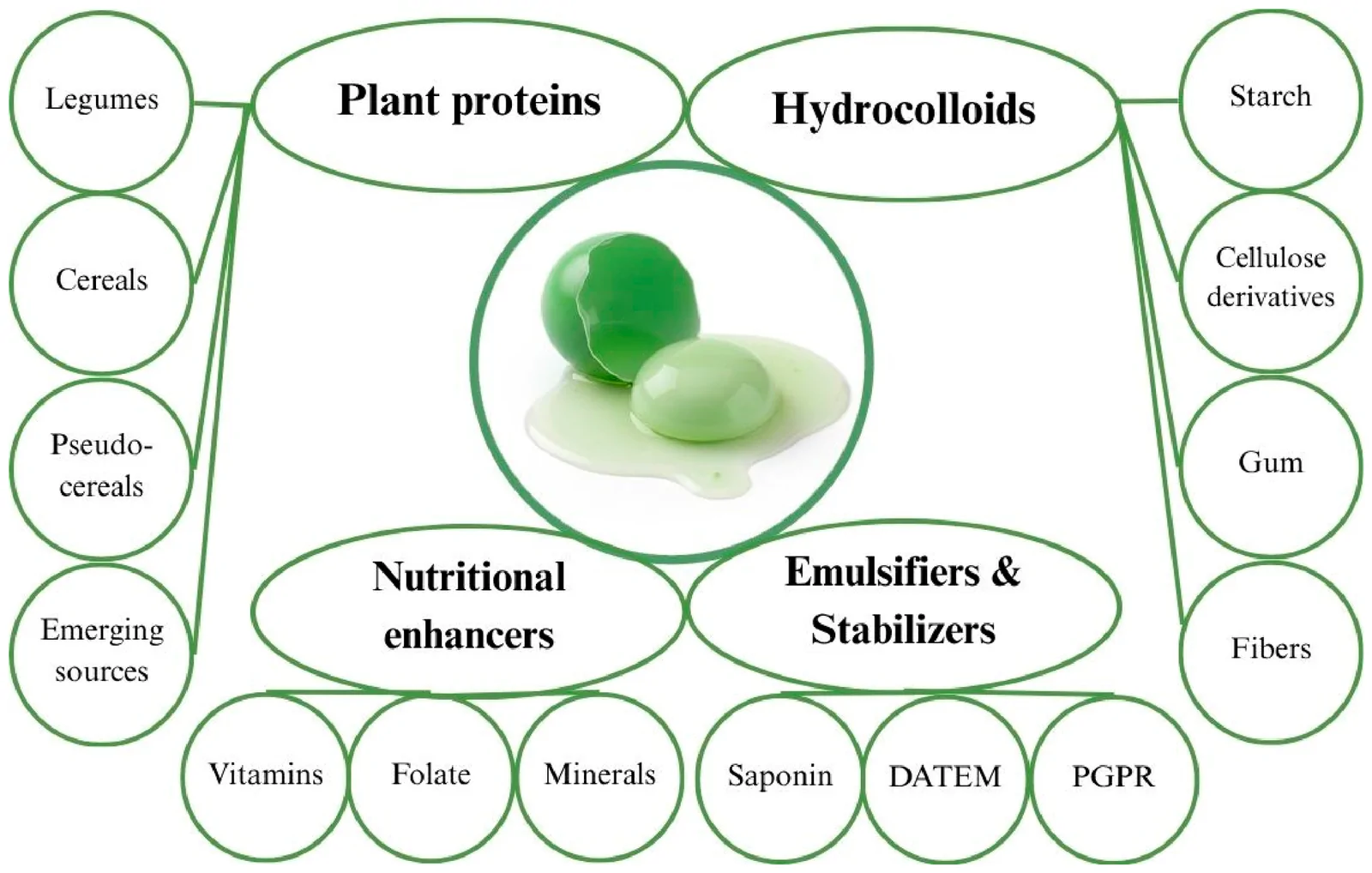
Main ingredients in egg alternative formulations.

Figure 2 illustrates the ingredients used in egg alternative formulations. Proteins are normally the backbone of all egg alternative formulations as replacers for egg white. These proteins can be sourced from legumes (e.g., mung bean, pea, chickpea) [32], soy [73], oilseeds [6], cereals [74], and other new sources, like microalgae [75] or duckweed [76]. Although all of these proteins differ in some regards from those of eggs, they can still confer structural and functional properties, such as coagulation, foaming, emulsification, and binding [4]. Depending on the food application and expected results, food additives can be added aiming to improve the techno-functional properties of the formulations, including xanthan gum, guar gum, modified starches, and dietary fibers [77]. Egg yolk alternatives are relatively more complex to prepare and formulate compared to egg white alternatives. This stems from their unique color, multilevel structure, rheology, and functionality. Generally, an oil mixture contains colorants, such as β-carotene [78], curcumin [79], lutein [32], plant extracts like mashed pumpkin [80], and any other generally recognized as safe (GRAS) pigments that can mimic the egg yolk color. The oil mixture is added to a pre-prepared aqueous dispersion of protein to develop a coarse emulsion prior to homogenization and the preparation of fine emulsions. The last step is to prepare an emulsion gel by the addition of protein powders and/or hydrocolloids, such as β-glucan, to the fine emulsion and prepare a gel with a similar protein content to conventional eggs [78]. It should be mentioned that there are several approaches to preparing emulsion gels. In addition to the above-mentioned method, gelled emulsions can be prepared by heating the emulsions containing proteins that can form thermally irreversible gels, such as RuBisCO [79]. Acid-induced gels can be another strategy in the case of using specific proteins, such as soybean proteins [81].

To formulate egg white alternatives, different protein sources, usually in their isolated form, are used at different proportions. In this step, researchers consider setting the protein content according to that of eggs (~10–11 g/100 g whole egg) [82,83]. The proteins are mixed with water containing one or more hydrocolloids to adjust the rheology of the solution and mimic the real egg white. In this step, adjusting the pH to a specific level to provide proper gels or a rheological profile is also important [83].

In the case of whole egg alternatives, the preparation of both egg white and egg yolk alternatives can be done separately, followed by the addition of the egg yolk alternative into the egg white alternative [80]. Other methods include the preparation of an emulsion-based system containing plant protein, plant oil, and a colorant that is heated to form gels, mimicking the yellowish color of a pre-mixed boiled egg [79].

### 3.3. Plant Proteins Used in Formulation of Egg Alternatives

#### 3.3.1. Legumes

There are multiple legumes that can be used as protein sources, such as pea, cowpea, chickpea, soy, mung, lentil, and adzuki. Adzuki is a legume containing 25% protein and considerable amounts of lysine (11.77–21.75 mg/g) and other essential amino acids (48.81–92.76 mg/g) [84] compared to common cereal grains. The higher lysine content makes it an ideal choice for supplementing plant food formulations based on cereal proteins to compensate for their lack of lysine [84,85]. This legume contains considerable sulfur-containing and hydrophobic aromatic amino acids, conferring on these proteins an excellent foam-forming ability, and thus, is useful to mimic egg white. In fact, adzuki protein isolate has a superior foam-forming ability in comparison with soy protein isolate [86]. The ability to form stable foams is associated with its lower surface hydrophobicity and higher free sulfhydryl content compared to those of soy protein isolate. These characteristics can positively affect the interfacial rearrangement and stiffness of the interfacial layer. Lee et al. [86] reported that using adzuki protein isolate as an egg white alternative could make meringue butter with a higher incorporation of air than soy protein isolate-based butter. Interestingly, the authors reported that the foam stability of adzuki protein isolate was considerably higher than that of real egg white proteins. Moreover, the amino acid profiles of these proteins provided by Sindhu & Manickavasagan [87] demonstrate that adzuki protein lacks tryptophan, an essential amino acid. This is not exclusively the case for this protein, prompting food scientists combine different plant proteins to prepare egg alternatives [80,88].

Apart from their isolate form, legume protein concentrates have been focused on as protein sources for egg alternatives [88]. Chickpeas, peas, soy, lentils, mung and broad beans isolates, and concentrates were used as egg replacers in pancake formulations by Bianchi & Simonato [88]. The researchers reported superior gel-forming ability fr the concentrates, as they needed lower concentrations (12–14%) to form gels, compared to their protein isolate counterparts (16–18%). This superiority was not only seen in their gelling properties, but was also revealed in their emulsion-forming capacity, foam-forming ability, and foam stability. Regarding the quality of the end products, egg protein and soy protein concentrates achieved the highest foam volumes of 98 and 89 cm^3^, respectively. Cowpea is another legume that can be used as an egg alternative. It was reported that the use of freeze-dried cowpea albumin provided a similar foam-forming ability and produced meringue cookies of similar microstructure compared to egg white [89]. Moreover, Choi & Hahn [90] previously compared cowpea protein isolate with commercial pea and mung bean protein isolates. The authors revealed that heat-induced gels made from cowpea protein isolate were stronger with higher water retention than the above-mentioned commercial protein isolates. Additionally, cowpea protein gels had a similar microstructure to those produced by real eggs. Moreover, the researchers reported that omelets prepared with commercial protein isolates formed a weak gel, and it could not resemble a real egg-based omelet structure. Although cowpea protein-based omelets could not completely resemble the real omelet, they were able to reinforce the robustness of the omelets and outperformed other commercial protein-based alternatives. Table 2 summarizes research on legume-based proteins used as egg alternatives. Overall, legumes show excellent functional properties, such as foaming, emulsifying, and gelling, which allow them to be used in a wide array of food formulations, including aerated desserts, sauces, and baked products.

#### 3.3.2. Cereals and Pseudo-Cereals

Studies on cereal proteins as egg alternatives are still limited compared to legume proteins. Most research focuses on wheat gluten proteins and oat proteins [33,94]. To date, the research on these proteins has explored mainly the creation of mixtures of alternative ingredients to meet specific food formulations rather than investigating individual cereal proteins in depth.

Cereals have functionalities like binding, thickening, and gelation for use as egg protein alternatives [33]. Since α- and γ-gliadins in gluten, barley proteins, and rice proteins have been shown to possess emulsification capacity, foaming properties, and freeze–thaw stability [33], their application as egg white and egg yolk alternatives can be promising. While several strategies have been adapted to modify or enhance the functional properties of gluten, its application might be limited due to not meeting the demands of consumers with celiac disease.

In the absence of gluten, oat grains contain approximately 20% protein, including a balanced amino acid profile and about 36% essential amino acids [94]. However, the limited use of oat proteins as egg alternatives might be due to their lower solubility, resulting in lower foaming properties and emulsification capacity in comparison to widely used proteins, such as pea protein [94]. Rice provides proteins rich in essential amino acids, such as methionine, with a high biological value (74%) and digestibility (93%); thus, they are considered sources of high-quality alternative proteins [95]. However, rice glutelin has low water solubility, especially at neutral pH, which limits its inclusion, as its techno-functional attributes are weak compared to other protein sources [95]. Similar limitations in functionality are the limiting factors for the application of proteins from cereals like barley [96] and corn germ albumin [97], despite the fact that their compositions are close to an ideal one. The nutritional quality of corn germ is comparable to that of egg and milk proteins [98].

Proteins from pseudocereals, such as amaranth [99], buckwheat [100], quinoa [101], and chia [102], have been widely studied in terms of their functionalities, nutritional value, and bioactivity, and their applications in alternative food products [103]. Mohammadi et al. [99] prepared egg yolk alternatives using amaranth protein isolate for formulation of low-fat mayonnaise. The authors reported that amaranth protein isolates showed high water (2.38 g/g) and oil (4.15 g/g) adsorption capacities and could provide stable emulsification capacity suitable for viscous food formulations. The application of chia seed protein hydrolysates as egg alternatives showed physical and thermal stability, firmness, and adhesiveness in alternative mayonnaise comparable to conventional mayonnaise [102]. The use of pseudocereal proteins in alternative food products has advantages and disadvantages in comparison to cereal-derived proteins. Pseudocereals possess higher or similar protein content compared to cereals, while the absence of gluten in pseudocereals provides another advantage, which facilitates their application in gluten-free diets [104]. Pseudocereals contain high amounts of methionine and cysteine, the limiting essential amino acids in legumes [4]. However, their cultivation is not as widespread as that of cereals, which might be due to the lack of awareness among many farmers, especially those in developing countries. It should be mentioned that due to increasing research on the excellent nutritional profile of pseudocereals, more farmers tend to add them to their cultivation lists [104].

At first glance, it might be perceived that limited functionalities restrict the use of cereal and pseudocereal proteins in egg alternatives in comparison to legume proteins. Therefore, it is crucial to highlight why it might be important to consider cereal proteins in egg alternative formulations. The first advantage of cereal proteins is their abundance. Cereals, particularly wheat and corn, are extensively used as starch sources worldwide, and therefore, the protein supplied as a secondary nutrient is widely accessible. Cereal proteins can also assist in balancing the amino acid profile of egg substitutes or alternatives. Legumes contain high amounts of lysine, while cereals generally provide more methionine, an essential amino acid that is limited in legumes [4]. Blending both cereals or pseudocereals with legumes can provide a more balanced or complete protein profile that can better improve the nutritional profile of the final products. Cereal proteins, such as rice protein, can be non-allergenic and gluten-free [95], which may appeal to consumers with various dietary restrictions or allergies. Cereal proteins often are flavorless and colorless [105] and can give egg alternatives or substitutes a more neutral taste and appearance, thereby increasing consumer acceptance. Regarding their functionalities, several applicable techniques such as fibrillation [106], chemical modifications [107], partial hydrolysis [105], fermentation [105], and extrusion [108] have been applied to modify cereal proteins. The modification techniques and the challenges in the application of cereal proteins are discussed in detail in Section 5 and Section 6.1, respectively.

#### 3.3.3. Emerging Sources of Proteins

Algae: Algae are a large group of photosynthetic eukaryotes, including macroalgae (seaweeds) and microalgae with about 200,000 species [109]. The application of algae in food formulation has begun in recent decades, but their use in alternative food products has started relatively recently, with most of the studies appearing since 2019 [110]. Among microalgae, *Arthrospira* spp. (formerly spirulina) and *Chlorella* spp. are supplied commercially and are known for their unique nutritional profiles. To the best of our knowledge, there are no published papers regarding the application of algae proteins for egg alternatives in Scopus. However, the use of algae has been explored in other alternative food formulations, especially meat analogs [109,111]. To show the potential of algae as sustainable and functional protein sources for egg alternatives, Table 3 presents recent research on algae proteins in other alternative food formulations. Studies have been retrieved from the Scopus database published by September 2025 using the keywords of “Algae” AND “protein” in their title, abstract, and keywords. For example, Sägesser et al. [111] reported that heterotrophic *Auxenochlorella protothecoides* contained 50% proteins/dry matter and might be comparable to soy protein concentrates and pea protein isolates for structuring meat analogs. The researchers reported that the texturizing effect of *A. protothecoides* may be attributed to its protein composition and properties rather than its macrocomposition. *A. protothecoides* protein was more soluble (16–68%) with lower hydrophobic side chains (15–50%), which may contribute to more intermolecular interactions and network formation in high-moisture extrusion (HME) cooking for meat analogues.

It should be mentioned that algae, as sustainable protein sources, have their advantages and disadvantages compared to other alternative protein sources. Microalgae/seaweed have extraordinarily high productivity relative to traditional plant crops, with a yield of 22–24 tonnes of dry biomass/ha per year compared to soybean and wheat [109,112]. In terms of protein yield per area, microalgae (4–15 tonnes/ha) and seaweed (2.5–7.5 tonnes/ha) outperform legumes like soya (0.6−1.2 tonnes/ha) [109]. Interestingly, some microalgal strains can even contain 78% protein, which is so much higher than soybeans (30–40%) [113,114]. In addition to quantity, their protein is of high quality with a balanced complement of essential amino acids. *Arthrospira* and *Chlorella* spp. have essential amino acid indices above 100 (102.6 and 107.5, respectively) and are comparable to casein from milk and higher than soya protein [109]. Nonetheless, microalgal proteins tend to have unbalanced amino acid profiles, and many species are deficient in lysine and tryptophan [78], which can restrict their use. The majority of brown algae, excluding a few examples, tend to be deficient in lysine, while many red algae are often modest in leucine and isoleucine [115]. In seaweeds, cysteine is invariably the most limited amino acid, while glutamic acid and aspartic acid are in good abundance [115]. These amino acid limitations may require the blending of algal proteins with complementary proteins from other sources in order to provide a balanced amino acid profile.

Insects and larvae: Insect-based ingredients are highly nutritious, and there are a wide number of edible insects in Europe, such as *Tenebrio molitor*, *Gryllodes sigillatus*, and *Schisocerca gregaria*, which contain ~52–76% protein with a complete essential amino acid profile [109,116]. The sustainability advantages of insect farming are striking. Insect farms use far less land and water and have much better feed-conversion ratios than conventional livestock. They emit far fewer greenhouse gases per kg of protein produced [116]. For instance, insects require only a fraction of the feed to produce the same protein as cattle, pigs or poultry, and they can be raised on organic side-streams [116]. In summary, insects have “lower water and land use, lower feed conversion ratios, and overall lower global warming potential” than conventional meat. Food scientists recently highlighted insect protein as a versatile ingredient for bakery goods [117], snacks [118], and meat alternatives [117]. Their use improves nutritional value by adding protein, fiber, and micronutrients as well as sustainability credentials. Insect proteins show promising egg-like functionality, as many studies report they can function as foaming, emulsifying, and gelling agents [119]. For example, it was reported that insect protein isolates from African migratory locust flours had foam-forming properties around 200% at pH 5 [119]. However, industrial manufacturers face challenges: insect powders can introduce strong flavors, a dark color, or extra oil, which may limit their inclusion levels. These challenges are not only related to their effect on the organoleptic properties of food formulations, but also stem from international legislation, a lack of a clear place in Western cuisine, consumer acceptance, food safety, and allergenicity [116].

Edible larval proteins, such as those from the larvae of *Aegiale hesperiaris* (maguey white worm) and *Comadia redtenbacheri* (maguey red worm), and mealworms [116,120], represent an innovative, highly sustainable, and nutritionally dense option for alternative proteins. They are rich sources of well-balanced amino acids and bioactive compounds, while also providing high digestibility to enhance the nutritional value of alternative formulations [121]. Similar to insects, larva protein production utilizes less land, water, and feed, relative to conventional protein sources, and thus has comparable environmental impacts [121]. Larva proteins have recently appeared in a variety of sustainable food products, like bakery products, snacks, and meat analogs, to improve their protein content and functionality [122]. Given their proven emulsification, foaming, and gelling properties, larva proteins also show promise as functional substitutes for eggs in various formulations, such as dressings [123] and eggless mayonnaise alternatives [124]. However, additional research on flavor optimization and regulatory approval is needed to harness larva proteins in mainstream alternative and hybrid food systems. Table 3 presents recent research on insect and larvae proteins in other alternative food formulations. Studies were retrieved from the Scopus database published by September 2025 with keywords of “insect” OR “larvae” AND “protein” in their title, abstract, and keywords. As shown, the majority of studies conducted so far have centered around the use of insect and larva proteins for meat analogs, while there have not been many studies examining alternative food formulations. Additionally, multiple aspects of their functional characteristics have not been explored. There is currently enough evidence to suggest that insect and larva proteins exhibit stable emulsifying properties, as well as antioxidant activity and thermal stability [125,126], which are important qualities for complex food formulations when eggs are replaced with sustainable sources of protein.

Fungi: Fungal proteins, particularly from fermentation using mycelium, yeasts, and molds, present an additional option for sustainable protein in alternative food systems. Fungi-derived proteins such as mycoprotein and edible mushrooms have recently gained popularity as promising protein options, as they contain high levels of protein (8–40%), a balanced amino acid profile, good bioavailability, and low environmental impacts [109,127]. Fungal proteins are not all alike, and there are multiple fungus types and species present. We can classify them into two general categories: First, mycoproteins derived from filamentous fungi which is usually a species such as *Fusarium venenatum* have been developed to produce mycoproteins which has high protein content and better texture [128]; and second, the mycelium that forms the vegetative parts of edible mushrooms, which have high contents of protein, dietary fibers, vitamins, and bioactive compounds. The structure of mushroom mycelia is naturally fibrous, and this can be utilized to develop new meat analogues and hybrid cell-cultured meat products [129].

Table 3 presents recent research conducted on fungal proteins in alternative food formulations. Studies have been retrieved from the Scopus database published by September 2025 with keywords of “fungi” OR “mycelium” AND “protein” in their title, abstract, and keywords. As shown, studies have focused on the production of meat analogs using mycoproteins as complementary ingredients in order to enhance the textural properties, water- and oil-holding capacities. According to Table 3, it can be inferred that fungal proteins can be used in egg alternative formulations as they can provide a good amino acid profile along with required functionalities such as increasing viscosity, gelation, and providing good in vitro digestibility. Studies showed that proteins derived from fungi are highly valued for their functionality in alternative food production, including improving the texture and nutritional value of alternative meat products [109]. For instance, a recent study performed by Zhang et al. [130] investigated the possibility of using *Penicillium limosum*-isolated protein as a co-ingredient in high-moisture meat alternatives and achieved large enhancements in viscosity, gelling property, chewiness, fibrousness, and in vitro protein digestibility (68.65%) by adding 5% mycoprotein. Products from the Quorn company with alternative egg also helped raise the profile of using biomass fungi as a direct substitute for meat in the United Kingdom. These products aim to capture the texture, flavor, and nutrition profile of meat through the generation of meat-like mycoproteins primarily from *Fusarium venenatum*. There are many ways to generate texture, including an array of gelling agents like agar, alginate, and egg albumin, but they must be processed to get to a meat-like product. During sensory analysis, these products were highlighted as having distinctive texture, taste, and appearance that meet consumer expectations for meat alternatives [128]. Despite their numerous advantages, there are several daunting challenges and limitations that must be resolved for fungi-based proteins to have broader acceptance [131]. There are foremost technical and production challenges in scaling-up production to industry levels, adequately developing meat-like products with regard to texture and taste, and achieving consumer acceptance [131]. There are also compliance and safety challenges such as food safety, new food regulations, and accurate labeling [131].

**Table 3:** Emerging proteins sources and their application in alternative food formulations.

Protein Sources	Alternative Formulations	Role in the Formulations	Functionality	**Reference**
**Algae (macroalgae and microalgae)**				
*Arthrospira platensis* *Haematococcus pluvialis* *Nannochloropsis oculata*	Meat	Complementary ingredient	↑ Nutritional quality ↑ Antioxidant capacity ↓ Hardness and chewiness by *A. platensis* ↑ Cut resistance and hardness by *N. oculata* ↑ Oil holding capacity by *H. pluvialis* ↑ Protein digestibility ↑ Nutritional and textural by *A. platensis* ↑ Visual and antioxidant properties by *H. pluvialis* ↑ Phenolic and chlorophyll contents by *N. oculata* ↑ Darkness	[132]
*Auxenochlorella pyrenoidosa* (Formerly *Chlorella pyrenoidosa*)	Meat	Complementary ingredient	↑ Springiness ↑ Chewiness ↓ Hardness ↑ Bound water content ↑ Digestibility ↓ Bitterness ↓ Astringency	[133]
*Arthrospira platensis*	Hybrid milk (*Arthrospira platensis* protein content 25%, 50%, 75%, 100%)	Complementary ingredient	↑ Greenish-brown hue ↑ Viscosity ↓ Thermal stability ↓ Gelation temperature	[134]
*Nostoc sphaericum*	Cheese	Complementary ingredient	↑ Nutritional value ↑ Acceptability	[135]
*Ulva fenestrate* *Palmaria palmata*	Whole biomass	Protein and Cobalamin (Vitamin B12) sources	Nutritional ingredients	[136]
*Pseudochlorella pringsheimii*	Protein buns	Fortification	↓ Softness ↓ Springiness ↑ Chewiness	[137]
*Nostoc sphaericum*	Alternative cheese	Fortification	↑ Acceptability	[135]
*Ulva lacinulata*	Protein-rich extracts	-	↑ Protein digestibility	[138]
*Chlorella vulgaris*	Microalgal single cell proteins	Multifunctional ingredient	↑ Gelling, ↑ Water holding capacity ↑ Solubility ↑ in vitro protein digestibility.	[139]
*Arthrospira platensis*	Pickering emulsion	Emulsifier	↑ Emulsion Stability	[140]
*Chlorella protothecoides* (Formerly *Chlorella protothecoides*)	Emulsion	Emulsifier	↑ Gel-like structure ↑ Emulsion stability	[141]
*Arthrospira platensis* *Chlorella* spp.	Chicken rotti	Fortification	↑ pH ↑ Lipids content ↑ Total amino acid content ↓ Some color parameters	[142]
**Insects and insect larvae**				
*Alphitobius diaperinus* Larvae *Acheta domesticus*	Meat	Stabilizer of high internal phase emulsions	↑ Protein aggregates by thermal treatment Elastic behavior ↑ Emulsion stabilization by thermal treatment	[143]
*Protaetia brevitarsis* Larvae	-	Complementary ingredient	↑ Antioxidant capacity ↑ Nutritional profile ↑ Essential amino acids The 7:3 ratio of larvae to isolated soy protein was the most effective	[144]
*Tenebrio molitor* (Brown mealworm; larvae)	Meat	Stabilizer	↑ Thermal stability ↑ Water holding capacity ↑ Gumminess	[145]
Back soldier fly (Larvae)	Meat Chicken breast	Complementary ingredient	↓ Hardness ↓ Cohesiveness ↓ Springiness ↓ Chewiness	[146]
*Alphitobius diaperinus* Larvae	Meat	Complementary ingredient	↑ Protein aggregates by thermal treatment ↑ Hardness ↑ Smooth network by CaCl_2_ addition	[147]
**Fungi**				
*Pleurotus eryngii*	Meat (Thai-Northern style sausage)	Complementary ingredient	↓ Hardness ↓ Chewiness ↓ Water holding capacity	[148]
*Fusarium venenatum*	Meat	Complementary ingredient	↑ Electrostatic interactions ↑ Overall texture properties	[149]
Shiitake mushrooms	Meat (Yak meat enema)	Complementary ingredient	No changes in cohesiveness and elasticity after storage (0, 2, 4, 6, 8 and 10 days at 4 °C)	[150]

The upward (↑) and downward (↓) arrows represent “high/increase” and “low/decrease”, respectively.

### 3.4. Hydrocolloids

Multiple hydrocolloids have been explored as egg replacers due to their ability to replicate the texture, structure, and functional properties of eggs, including their recent use in alternative formulations. Hydrocolloids, such as κ-carrageenan, gellan gum, and β-glucan, interact with plant proteins to exhibit gel and foam characteristics that imitate the texture and structure of eggs. The interactions between hydrocolloids and plant proteins may involve electrostatic, hydrogen-bonding, hydrophobic, and other non-covalent interactions, depending on the chemical characteristics of the polymers and the environmental conditions. The concentrations of hydrocolloids and their interactions with proteins can be adjusted to control the hardness, gel strength, and specific volumes of egg analogues [32].

For egg alternatives, it is essential to replicate the binding and gelling action of natural eggs. The structure of egg products, including scrambled eggs and omelets, relies upon the thermoreversible gelling capability of hydroxypropyl methylcellulose and methylcellulose [151,152]. These hydrocolloids behave as liquids at ambient temperature, allowing the product to be poured from the container easily, but they set into stable gels when heated. The gelling behavior effectively mimics the coagulation of egg protein. This thermal property allows the products to be cooked and handled in the same manner as traditional eggs [151]. There are also other gelling properties for cold or baking uses. Different types of hydrocolloids or gelling agents can be used, and a few examples include agar, sodium alginate, carrageenan, and pectin, which can create solid structures, like those in custards or desserts [33].

Emulsification has an integral function in producing a rich mouthfeel and stable texture in egg alternatives and baked goods. While plant proteins, such as mung and fava bean proteins, have some emulsification properties, hydrocolloids are often necessary to provide additional stabilization of the emulsion and control of its rheological properties [153]. For example, it was reported that the emulsifying activity and stability of protein-based systems can be significantly improved by the addition of xanthan gum and kappa-carrageenan into formulations [4]. However, the mechanisms by which hydrocolloids improve emulsion stability should not be attributed solely to enhanced interfacial protein adsorption. Many hydrocolloids are predominantly non-adsorbing or weakly surface-active and can stabilize emulsions by increasing the viscosity of the continuous phase, thereby reducing droplet movement and collision, and by providing steric or physical stabilization. In addition, hydrocolloid–protein interactions can modify protein conformation, interfacial adsorption, and the properties of the interfacial layer under specific formulation conditions. Thus, the improvement in emulsifying activity and stability observed after hydrocolloid addition depends on the specific protein–hydrocolloid system, polymer concentration, pH, ionic strength, and processing conditions [32,154]. Not all hydrocolloids are used for this same function. While the use of xanthan gum and carrageenan has been reported to be useful to improve emulsification, the use of guar gum, on the other hand, has been found to have the opposite effect [154]. This clearly indicates the need for precise ingredient selection and formulation, as a “gum blend” is not suitable in every situation.

Aeration or foaming potential is one of the most difficult characteristics to mimic when replacing eggs, especially in an alternative formulation. Hydrocolloids, while not generally considered principal foaming agents, act most importantly as stabilizers. Some studies have shown that xanthan gum and guar gum can greatly increase the stability, uniformity, and density of foams. For instance, xanthan gum is a popular ingredient to add to liquid mixtures because it increases foaming stability and can trap air during mixing to give volume and a light texture [154], which is particularly useful for products that are expected to be soft and airy, like cake and mousse. An example of this principle in a natural system is aquafaba, the liquid contained in canned chickpeas. The foaming ability of aquafaba is due to the fact that it contains a variety of natural hydrocolloids, including starches and proteins, which together provide the structure and the stability to the foam [155]; and thus, it can be used as an egg substitute in alternative meringues and sponge cakes [83,156]. To provide an overview of these hydrocolloids, recent studies on their applications, roles, and functionalities in egg alternative formulations and products are presented in Table 4.

### 3.5. Starches and Fibers

Starch plays an important role in the formulation and functionality of egg alternatives, as it is capable of influencing the texture, structure, and nutritional aspects. Starch can be used to effectively mimic the gelling, thickening, or binding properties of eggs. Different starches (corn, glutinous rice, potato), when mixed with plant proteins, such as soy protein isolate, can be customized to find suitable textures and volumes comparable to real eggs [157]. Lu et al. [157] reported that soy protein isolate/potato starch at 8:6 improved the hydrogen and ionic bonding and gel structure while decreasing its in vitro digestion. These mixtures allow proteins to create and develop a good gel network, replicating the mouthfeel and structure of eggs. Starch can be modified physically, chemically, or enzymatically to enhance its functional properties, such as swelling, viscosity, and gel formation [158]. Modified starches can be used as thickeners, stabilizers, and gelling agents in alternative foods, including egg analogues [159,160]. It appears that encapsulation systems based on starch may have the potential to deliver flavors, nutrients, and bioactives promptly and thus increase the sensory and nutritional profile of egg alternatives. To the best of our knowledge, no research has been published in this area thus far. Table 4 provides the recent research on the application of starch as functional ingredients in egg alternative formulations and products. As shown, despite their diverse functional properties, there is limited research on starch in comparison to the other hydrocolloids, indicating that more studies on the applications of starch and its derivatives for egg alternatives and egg-based products are required.

### 3.6. Non-Protein-Based Emulsifiers and Stabilizers

In practice, combinations of non-protein plant-derived stabilizers and emulsifiers are used to stabilize oil-in-water emulsions, adjust viscosity and texture, retain moisture, improve mouthfeel, and extend shelf-life. For example, hydrocolloid gums like xanthan, guar, etc., can bind water and thicken the liquid matrix, while plant lecithin and other emulsifiers ensure fat dispersion [33]. By tailoring these additives, alternative egg products such as liquid egg substitutes or egg-free bakes can achieve egg-like creaminess and stability. Notably, egg alternatives often stay fresh longer and resist syneresis, since added gums slow staling and moisture migration [33]. Lecithin extracted from soy, sunflower, rapeseed, corn, rice bran, cottonseed, and camelina seed, as well as mono- and diglycerides, diacetyl tartaric ester of monoglycerides, polyglycerol polyricinoleate, and Quillaja (soapbark) saponin is among the popular emulsifiers for developing novel food products [4,161,162]. Inulin, psyllium husk fiber, β-glucan from cereals, cellulose fiber, and carboxymethyl cellulose are among the fibers and thickeners that can be applied as stabilizers for egg alternatives [4,161,162]. Table 4 summarizes the non-protein-based emulsifiers and stabilizers recently used in egg-free food formulations. As an example, Tay et al. [163] explored the application of soy lecithin for stabilizing an emulsion prepared with amaranth protein and its application for alternative omelets. The addition of this alternative lecithin strengthened disulfide bonds upon heating. The promotion of these interactions could positively influence the structure of alternative omelets.

**Table 4:** Hydrocolloids used as egg alternatives.

Additives	Application in Alternative Formulations	Role in Formulation	Functionality	Reference
**Hydrocolloids**				
Locus bean gum (in form of foam-mat dried) Fava bean aquafaba (in form of foam-mat dried)	Muffin	Whole egg alternative	Maximum foam overrun (832.37%) for aquafaba ↑ Foam capacity of proteins by increasing drying temperature Maximum foam capacity for fava bean aquafaba powder ↑ Foam stability by the addition of locus bean gum compared to aquafaba alone. ↓ Foam drainage by the addition of locus bean gum compared to aquafaba alone.	[164]
Xanthan gum Lysozyme nanoparticles Konjac glucomannan	Mayonnaise (Pickering emulsion)	Egg yolk alternative	↑ Network structure by konjac glucomannan up to 40% substitution compared to yolk alone and other replacement ratios. ↓ Oil droplet aggregation by konjac glucomannan compared to yolk alone and other replacement ratios. ↓ Droplet size by konjac glucomannan compared to yolk alone and other replacement ratios. ↑ Centrifugal stability by incorporation of xanthan gum, lysozyme and especially konjac glucomannan than egg yolk alone.	[165]
Guar gum Sodium alginate	Sponge cake	Egg white alternative	↓ Foam forming ability by increasing guar gum compared to sodium alginate No change in foam forming ability by increasing sodium alginate concentration No predictive trend in foaming properties after changing hydrocolloid concentration	[83]
Corn fiber gum-zein-lecithin particle	Mayonnaise (High internal phase emulsions)	High internal phase emulsions as whole mayonnaise	Acted as an emulsifier Acted as a surfactant ↑ Molecular rearrangement at the oil-water interface due to the incorporation of zein-corn fiber gum-lecithin particles compared to zein particles and zein-corn fiber gum particles. ↓ Tribological property compared to egg-based mayonnaise ↑ Creaminess compared to egg-based mayonnaise ↑ Smoothness compared to egg-based mayonnaise ↑ Physical stability compared to egg-based mayonnaise ↑ Thermal stability compared to egg-based mayonnaise ↓ Digestible fat in the small intestine compared to egg-based mayonnaise	[166]
Chia Seed gum Flaxseed gum Rocket seed gum	Mayonnaise	Whole egg and fat alternative	Rheological properties, zeta potential, and particle size were similar to those of guar gum, Arabic gum, and xanthan gum-based mayonnaise	[167]
Xanthan gum Chickpeas aquafaba (treated by citric acid and salt)	Cake	Egg white alternative	↑ Foaming capacity by pre-treated chickpeas aquafaba compared to xanthan gum ↑ Foaming stability by pre-treated chickpeas aquafaba compared to xanthan gum ↑ Foaming capacity and stability by the addition of xanthan gum compared to xanthan gum ↑ Viscosity of the batter by the addition of xanthan gum compared to xanthan gum Unchanged hardness of cake Different properties to control cake	[168]
**Starch and fibers**				
Octenyl succinate starch (OSA-Starch) Complemented by sodium caseinate and tannic acid	Cake (Hybrid) Ice Cream (Hybrid)	Whole egg alternative (partial replacer)	↑ Specific volume of cakes compared to whole egg-based cake ↑ Hardness of cakes compared to whole egg-based cake ↓ Elasticity of cakes compared to whole egg-based cake ↑ Chewiness of cakes compared to whole egg-based cake ↓ Melt resistance of ice cream compared to whole egg-based ice cream ↑ Hardness of ice cream compared to whole egg-based ice cream ↓ Viscosity of ice cream compared to whole egg-based ice cream	[159]
Corn starch Octenyl succinic anhydride (OSA) corn starch	Mayonnaise	Egg yolk alternative	↓ Fat content by 41.7% compared to egg yolk-based mayonnaise Obtained scored 3 by the semi-trained panelists	[160]
**Non-protein-based emulsifiers and stabilizers**				
Diacetyl tartaric acid esters of monoglycerides (DATEM)	Gluten-free cake	Emulsifier	Optimum formulation: 13.3% sprouted mung bean flour 0.615% Plantago mucilage 1.2% DATEM emulsifier 60% grape syrup	[169]
Aquafaba Flaxseed	Mayonnaise	Emulsifier	25% aquafaba and 5% flaxseed resulted in: 99.99% Emulsion stability Neutral to favorable scores for taste, color, aroma, texture, and overall acceptability	[170]
Modified rice starch granules	Mayonnaise	Pickering emulsion stabilizer	Formulation: 200 mg/mL pre-gelatinized rice starch, 50% (*w*/*w*) olive oil and 50% (*w*/*w*) lemon juice resulted in: Emulsion index of 100% Highest emulsion stability Smaller droplet size compared to emulsions stabilized by native and OSA rice starch Longer storage stability compared to emulsions stabilized by native and OSA rice starch	[171]
Defatted soybean powder treats with supercritical carbon dioxide	Mayonnaise	Emulsifier	↑ Emulsion stability compared to soy flour-based mayonnaise ↑ Viscosity compared to soy flour-based mayonnaise ↑ Particle size uniformity compared to soy flour-based mayonnaise ↑ Sensory properties compared to soy flour-based mayonnaise ↓ Particle size compared to soy flour-based mayonnaise	[172]
Red ginseng extract	Mayonnaise	Emulsifier	↑ Antioxidant activities compared to egg-based mayonnaises ↓ Particle size compared to egg-based mayonnaises ↑ Viscosity compared to alternative mayonnaises with higher substitution rates (>25%) mayonnaises with higher substitution rates (>25%) ↑ Emulsion stability and alternative mayonnaises with higher substitution rates (>25%) ↓ Emulsification properties by higher extract content (>75%)	[173]
Xanthan gum Modified starch	Mayonnaise	Emulsifier	Similar viscosity, stability, and firmness compared to commercial products	[174]
Chickpea aquafaba treated with ultrasound	Mayonnaise	Emulsifier	↓ Oil droplet size compared to egg yolk-based mayonnaise 81.30% Emulsion stability by 30 min ultrasound treatment ↑ treatment time resulted in ↓ emulsification properties compared to egg yolk-based mayonnaise	[175]
Korean-grown soy and chickpea aquafaba	Comparison between cooking without and with presoaking	Emulsifier	Aquafaba with presoaking produced better emulsifiers: 90% Emulsion stability	[176]

The upward (↑) and downward (↓) arrows represent “high/increase” and “low/decrease”, respectively.

### 3.7. Nutritional Enhancer Ingredients

As noted above, the protein in alternative eggs falls short of real eggs when it comes to amino acid composition and overall nutritional value. Food scientists tend to formulate egg alternatives by complementing proteins from different sources to mimic the amino acid profile of eggs. However, there are several other nutrients, such as vitamins, minerals, fats, and other compounds that have to be considered when evaluating the nutritional quality of plant-based egg alternatives.

Eggs are high in micronutrients, including omega-3 fatty acids, vitamins like A, D, E, B_12_, B_2_, B_5_, folate, and choline, and minerals such as iron, phosphorus, and selenium [4]. Comparatively, plant foods generally contain insignificant amounts of these micronutrients, especially B12 and D [32]. Studies on alternative diets revealed lower intakes of vitamin B12, vitamin D, iron, zinc, calcium, and iodine compared to animal-based diets [177]. Food scientists and technologists have proposed fortification as a solution to address these nutritional gaps [78,178,179]. Nutritional models suggest that adding critical micronutrients such as vitamins B_12_ and D, calcium, and iron to alternatives can help achieve dietary targets [177]. Also, it should be noted that the digestion and bioavailability of micronutrients in alternative food products must be similar to their animal-based counterparts, as deviations can raise concerns about human nutrition and well-being. For this purpose, S. Li et al. [78] studied the protein and lipid digestion behaviors and vitamin D in vitro bioaccessibility in egg yolk replacers. The researchers found a similar free fatty acid content in the small intestine compared to real egg yolk. It was also reported that the increase in polysaccharides could decrease lipid digestion, decreasing health concerns about lipid intake of alternative egg yolks. However, the increase in polysaccharides impacted the bioaccessibility of vitamin D negatively. To address this issue, further studies are required to investigate the effects of other polysaccharides on the bioaccessibility of vitamin D in alternative egg yolks.

Moreover, further research has improved the nutritional properties of egg alternatives and enhanced their health benefits. Vu et al. [178] fortified egg alternatives with lutein, aiming to improve eye health. This carotenoid exists naturally in real eggs and can reduce age-related macular degeneration. Due to its color properties, it could also introduce a red to yellowish color to egg alternatives. The researchers found that long-chain triglycerides could increase lutein bioaccessibility due to the rapid and complete digestion of these lipids. The results showed that long- and medium-chain triglycerides could act as carriers to improve the bioaccessibility of lutein by around 80%.

## 4. Commercial Products

Commercial egg alternatives are currently marketed in various forms, including liquid preparations, powders, frozen ready-to-use products, and specialty baking products, thus fulfilling both domestic and commercial demands. Table 5 summarizes key companies currently producing egg alternatives, among which the best-known product is JUST Egg (Eat Just, Inc., Alameda, CA, USA), almost entirely consisting of mung bean protein [70]. The product is highly utilized as a scrambled egg replacer and has gained high market presence due to its capability of closely resembling cooked eggs in terms of texture, color, and flavor while being cholesterol-free. JUST Egg offers egg replacers as liquid and pre-cooked products, which are convenient to use for daily meals in household and foodservice facilities. Likewise, Zero Egg (Israel/USA) has been developed as an all-purpose alternative to eggs for cooking and baking, including specialty versions tailored for foodservice and commercial kitchens [71].

In Asia, products like OnlyEgg (Float Foods, Singapore) are innovations that move beyond traditional scramble substitutes. OnlyEgg has developed a legume-based “whole egg” that is visually and texturally similar to the separation of yolk and white, to enable cooking like sunny-side-up or soft-boiled eggs [72]. Beleaf (City of Industry, CA, USA) is also selling an alternative “sunny-side-up” egg for foodservice establishments, which expands the menu possibilities of plated meals [69]. Other types of commercial egg alternatives made in Europe include Tamalga from Algama Foods (Paris, France). Tamalga is a powdered whole-egg substitute made from microalgae proteins, mainly for the bakery and pastry sectors. This product highlights shelf stability and functionality like emulsification, foaming, and binding, which makes it versatile for larger food manufacturing companies [180].

Current commercial egg alternatives have demonstrated substantial advances in their functional properties and their ability to replicate the technological performance of conventional eggs across a range of food applications. Nevertheless, variability remains in sensory acceptance, cost, nutritional quality, and functional performance among available products. These developments reflect continued innovation in the quality and availability of egg alternatives while highlighting the need for further research to improve their functional performance, nutritional value, sensory characteristics, and cost-effectiveness.

## 5. Innovations and Future Perspectives

### 5.1. Improving Texture and Other Sensory Properties

Enhancements of texture, rheology, and other sensory properties of egg alternatives are regularly explored by modulating their gel structure with the ultimate goal of replicating real eggs through food processing.

Recent developments in this field include the use of customized emulsion-gel structures to create egg texture and appearance. For example, Zhou et al. [79] created an oil-in-water emulsion using RuBisCO protein that can self-assemble into a gel by heating. The researchers manipulated the droplet size of oil to control the color and firmness of the gel, where smaller droplets produced a more white, firmer gel and larger droplets created a softer, more yellow gel to mimic the behavior of cooked egg white. Curcumin was also incorporated into the oil phase by pH-driven encapsulation to replace the golden yolk color. In this study, it was found that droplet size is a key factor in controlling the texture of the egg alternative, so that emulsion gels with small (≈0.15 μm) and medium (≈0.74 μm) droplet sizes resembled the real egg’s hardness (≈2000 g), and chewiness (≈500), and springiness (90%) compared to the gels with larger droplet sizes (≈15.17 μm). The findings were attributed to the active-filler effect of the oil droplets. Large droplets could not fit effectively within the pores of the developing protein network and therefore disrupted it. Smaller protein-coated droplets, however, could be incorporated into the network and interact with it as active fillers, reinforcing the three-dimensional protein gel structure. Regarding the cooking behavior, the alternative egg showed a steep increase in complex shear modulus (G*) at approximately 45–65 °C, whereas real egg exhibited its major increase around 75 °C. The authors therefore conclude that the RuBisCO formulation would cook faster than conventional egg. This difference might originate from the lower thermal stability of RuBisCO. DSC showed a major denaturation peak around 66 °C for the PB egg, compared with approximately 61 °C and 81 °C for the ovotransferrin- and ovalbumin-associated transitions in real egg. Interestingly, after cooling, the G* values of the two systems became similar, suggesting that their final cooked mechanical properties could be relatively similar despite the different cooking temperatures. The differences in rheological behavior may have implications for sensory perception; however, this needs to be established in future experiments.

Another approach includes the use of dual-network, namely “bigel”, structures made of plant-protein hydrogels and structured oils, which are known as oleogels. For example, pea-protein hydrogels were mixed with candelilla-wax oleogels and cross-linked with microbial transglutaminase (TG) by Friedman et al. [181]. The protein–oleogel mixture (~10% TG) formed a stable gel, and the authors manipulated the oil: water ratio to tune the consistency and mouthfeel of the gels. The study demonstrates that bigel texture is highly tunable. Using 10% transglutaminase out of the protein mass yields a strong, resilient gel, but increasing transglutaminase to 15% or 20% creates a deformed, brittle texture with deep cracks. Adjusting the oleogel-to-hydrogel (OG/HG) ratio to 40/60 produces maximum hardness, while ratios of 50/50 and 60/40 shift the matrix into a bi-continuous structure with a “spreadable margarine-like behavior”. Furthermore, adding salt (NaCl) drastically reduces hardness and cohesiveness, transitioning the gel from a flaky, brittle solid to a much softer, smoother, and spreadable material. This structural flexibility allows formulators to target specific egg formats. The thermal rheology tests reveal a unique temperature response. As the bigel heats, it softens moderately up to about 50 °C, followed by an abrupt loss of firmness as the wax oleogel melts. However, as temperatures rise above 70 °C, the storage modulus (shear resistance) sharply increases again. This late-stage firming is driven by the pea protein reaching its denaturation point, exposing hydrophobic regions and causing thermal aggregation. Notably, the presence of salt actually enhances this high-temperature firming due to a charge-screening effect that favors these hydrophobic interactions. According to these findings, hybrid hydrogels and bigels can provide tunable oil-and-water structures that can be formulated to closely resemble the multi-textured mouthfeel of eggs.

Regarding sensory properties, there have been diverse techniques to replicate the flavor of real eggs. It should be noted that mimicking the cooked flavor of real eggs is not always feasible. For example, using egg white and yolk alternatives in alternative custard or mayonnaise is generally not accompanied by a cooked flavor, but it is a requirement when egg alternatives are used in bakery products. It has been reported that Himalayan black salt can provide a sulfur taste to food formulations, such as scrambled eggs, due to its high sulfur concentration [33]. Also, Khalifa et al. [33] proposed the use of dehydrated onion or garlic to mimic the cooked flavor of real eggs.

### 5.2. Improving Functional Properties

Functional properties are critical to the formulation of egg substitutes, including emulsification, binding, foaming, leavening, and coagulation properties. Enzymatic modification of proteins and hydrocolloids, like starch, can also be regarded as a method to modify the foam-forming ability [182] and emulsification properties [158], as well as to enhance the bioavailability of the original compounds [183]. Enzymatic crosslinking, for example, can dramatically alter the functionality of the compounds. Through this process, potato proteins crosslinked by fungal laccase treatments significantly improved protein solubility, emulsification properties, and foaming capacity, whereas extensive oxidative crosslinking decreased the molecular flexibility of the proteins, reducing their functionalities [184].

Conjugation can also be regarded as an innovative approach to enhance the functionality of proteins and hydrocolloids in egg alternative formulations. In a study, Sahraeian et al. [185] introduced the protein–polyphenol, polysaccharide–polyphenol, and protein–polysaccharide–polyphenol conjugates as multifunctional ingredients with diverse applications. The researchers claimed that esterification of polyphenols can provide significantly higher surface activity, gel properties, and emulsification activity along with antioxidant and antimicrobial properties for proteins and polysaccharides. These functionalities, along with higher bioavailability of bioactive compounds, might step up toward an advanced approach to formulating egg alternatives.

Many plant proteins from legumes and cereals partially mimic gelation, foaming, emulsification, binding, thermal stability, mouthfeel, and other required functionalities in egg alternative formulations, but often have drawbacks like poor gel strength, off-flavors, allergenicity, incomplete amino acid profile, solubility issues, etc. Although blending these proteins might be a viable option, further research is still needed to use proteins from other sources to provide adequate functionalities of the protein mixture in formulations while maintaining sustainability. Novel proteins extracted from other crops like pseudocereals [99], aquatic plants [79], microbial cells [186], and genetic engineering or molecular farming approaches are amongst the most recent research efforts to supplement proteins with higher functionalities.

### 5.3. New Processing Technologies

Novel and emerging processing methods let formulators overcome two major limits of alternative foods: (1) plant proteins and carbohydrate matrices often lack the native structure and multi-functionality of animal proteins, such as gelation, emulsification, foaming, and fibrosity [187]; and (2) some desirable attributes such as flavor, color, nutrient profile, and shelf life are harder to achieve with conventional processing alone [188].

Technologies, such as HME, non-thermal processing approaches such as pulsed electric field (PEF), cold plasma, high-pressure processing (HPP), ultrasound, microfluidization, 3D food printing, precision fermentation, and encapsulation provide complementary routes to produce alternative food products with improved sensory and functional performance while addressing safety and sustainability goals. Several reports also indicated that cold plasma could enhance the functionalities of proteins and polysaccharides, making them suitable ingredients for alternative food formulations [189,190].

Recently, Kim and Min [191] applied plasma-activated water to transform soy protein isolate into high-performing egg white substitutes by altering its molecular structure, interfacial behaviors, and thermal stability. Native soy proteins feature compact globular structures that bury hydrophobic residues, causing poor foam capacity and rapid collapse when whipped or baked. Plasma-activated water treatment generates reactive oxygen and nitrogen species that disrupt hydrogen-bonding networks, increasing the surface negative charge (zeta potential from −11.2 mV to −29.0 mV) and exposing buried sulfhydryl groups and hydrophobic sites required for rapid adsorption at the air–water interface. This molecular flexibility converts the foam phase from a weak liquid into an elastic, solid-like gel network, increasing the storage modulus (G′) from 5.8 Pa to 215 Pa, boosting air overrun, and drastically reducing liquid drainage. Furthermore, combining plasma-activated water with citric acid drives controlled disulfide re-crosslinking into fine, uniform micro-aggregates (~290.4 nm), achieving a 508% foaming capacity that matches natural egg white while preventing thermal collapse during baking to produce meringues with natural lightness, minimal yellowness, and realistic mechanical hardness. HME processes, including careful control of temperature profile, shear, water content, and die geometry, are applied to produce continuous, homogeneous protein gels with tailored bite and minimal fibering for folded egg products. Rolandelli et al. [192] reported that extrusion of rice starch underwent thermomechanical changes which finally led to fibrillar structures suitable for alternative foods with a juicy and soft texture.

Improving microbial safety, decreasing undesirable enzyme activities, degrading mycotoxins, and degrading pesticides are other applications of cold plasma processing in alternative food production [193]. Mild thermal and staged heating strategies can be regarded as a novel processing method to enhance egg alternative functionalities. Li et al. [194] reported that microwave-heated foams created by faba bean protein isolate at pH 4 showed increased protein aggregation and merged air bubbles in comparison to unheated egg white and faba bean protein isolate. This revealed the importance of staged heating of egg alternatives when incorporated into alternative food formulations.

Fermentation and cell-based processing are emerging technologies that can be applied to the production of egg alternatives. Two processing vectors are noteworthy: (a) controlled microbial fermentation of plant protein slurries that can improve flavor, solubility, and functional properties; and (b) precision fermentation to produce functional ingredients, such as recombinant albumins and surfactants that are blended into plant matrices to confer specific functionality [195].

3D printing is positioned as a complementary process to other structuring technologies, such as extrusion and fermentation. For egg alternatives, promising short-term research directions include: (1) Development of coaxial core–shell inks that can reliably print lipid yolk and protein white and set during mild post-print heating; (2) printable formulations leveraging fractionated albumin-like proteins to improve coagulation kinetics; (3) in-line enzymatic crosslinking modules to tune texture post-deposition; and (4) techno-economic analyses of multi-nozzle, parallelized printing farms for niche premium markets like custom nutrition, hotel/restaurant, and institutional feeding [196]. Recent studies provide protocols and rheological targets useful for researchers translating egg-analogue formulations to printable inks [196,197].

## 6. Current Challenges and Proposed Solutions

While plant-based egg alternatives are frequently promoted as more sustainable than conventional hen eggs, these claims require rigorous quantitative comparison using Life Cycle Assessment (LCA) methodologies. Evaluating environmental performance necessitates defining a precise functional unit—such as a mass-based unit of 100 g of product or a protein/function-adjusted equivalent—to accurately compare socio-economic and environmental impacts across the supply chain. When assessed holistically, the environmental benefits of alternative protein systems are not absolute; they depend heavily on the processing intensity required to replicate egg functionality. For example, while raw plant ingredients may have a lower initial carbon footprint, the energy requirements for intensive plant protein isolation, extraction, and extrusion can significantly elevate the cumulative energy demand and overall environmental burden. Furthermore, from an economic perspective, these intensive processing requirements inflate ingredient costs. As a result, commercial prices for plant protein egg alternative powders currently retail at a significant premium per functional unit compared to commodity native egg powders. Therefore, future product formulations must optimize processing energy efficiency to ensure alternative systems are genuinely more sustainable and economically competitive than the animal-based systems they aim to replace.

### 6.1. Technological Challenges

A central challenge in the advancement of egg alternatives is mimicking the multifunctionality of eggs. This functional gap can create challenges in performance, particularly in multi-component systems, such as baked goods, where eggs provide structure, aeration, and moisture retention. Recommendations for solving these functionality issues include formulating ingredients in combinations of proteins with other proteins, polysaccharides, lipids, or enzymatic crosslinkers. Additionally, there is an increasing interest in biotechnological approaches to building precision fermented, recombinant egg proteins for specific functionalities that are otherwise absent from plants.

Another immediate but pressing technological challenge is scaling up the production of egg alternatives at competitive costs. Often, alternative protein processing requires extensive refinement such as fractionation, purification, and functionalizing to create valuable end-products, which can drive up costs. Not to mention, variables arise, such as ensuring that final products comply with standardization based on the quality of the original raw materials, which can be impacted by crop variety, seasonality, and the geography where it was grown. Possible solutions include the optimization of primary plant proteins from the upstream growing and supply chain, achieving mild and effective extraction processes, and/or implementing scalable processing methods, such as HME and enzymatic processing. Moreover, the use of digital process control and process analytical technologies could enhance standardization and reproducibility at an industrial scale, and thus improve the efficiency and cost competitiveness.

### 6.2. Nutritional Challenges

Despite the fact that many egg alternatives closely resemble their analogs in appearance and functionality, achieving the same level of nutrition can be challenging. Eggs contain high-quality protein, choline, and essential micronutrients including vitamins A, D, E, and B_12_, as well as minerals like selenium and iron. Most alternative proteins, on the other hand, are missing one or more essential amino acids, and suffer from a deficiency of vitamins B12 and D, which are barriers to any vegan formulations. These nutritional gaps can be filled with nutritional fortification techniques, such as using vitamin and mineral premixes, omega-3 fatty acids, and protein complementation, such as mixing legumes with pseudocereals. As biofortification of crops continues to advance through genetic engineering and/or breeding, there can hopefully be improvements in nutritional equivalence.

Another important nutritional issue is allergenicity. Products containing common protein sources, such as soy and wheat gluten, rank among the top allergens, raising both health and marketing concerns. Points of consideration can be to use different, low-allergen protein sources like mung bean, faba bean, or potato protein, or microalgal protein, as well as to reduce allergenic epitopes through protein modification or fermentation. In addition, transparency in labeling allergens and educating consumers can reduce risks of allergenicity while improving trust in alternative products.

### 6.3. Consumer Acceptance

Even when technological and nutritional issues are addressed, consumer acceptance is another critical bottleneck. Many consumers view egg alternatives as inferior in taste, texture, and overall sensory attributes compared to real eggs for formulations in which they are the main ingredient, such as scrambled eggs. Suggested actions include formulation optimization informed by sensory design; flavor precursors and natural volatiles used to emulate the top note of egg; and structured fats and hydrocolloids used to promote mouthfeel. Sensory panels, as well as consumer co-creation strategies, can direct iterative development in alignment with consumer preference. Cultural and psychological determinants can also impact acceptance, as eggs form the basis of many cuisines and are deeply embedded in culinary customs where consumers may deem “alternative” as unnatural or over-processed. To address barriers to acceptance, transparent communication about the health, ethical, and environmental advantages of eating egg alternatives is vital to successful outcomes. Lastly, marketing strategies highlighting the natural ingredients found in egg alternatives, as well as clean-label formulations with sustainability credentials, would increase willingness to try egg alternatives.

### 6.4. Regulatory and Labeling Issues

The production and commercialization of egg alternatives must occur within the context of food safety regulatory frameworks, which differ by region. Each of these regulatory contexts comprises different challenges, such as adequately demonstrating the safety of novel ingredients (e.g., some proteins from fermentation or algae), obtaining GRAS status in the United States, or passing novel food approval in Europe. There is an ongoing need to develop and standardize testing protocols for functionality and safety. Collaboration with regulatory agencies in a preemptive manner can provide faster approval while building confidence. In addition to regulatory complexity, labeling and marketing also add another layer. Regulatory standards restrict the use of established and traditional nomenclature such as “egg” and “egg substitute” for products that do not contain any animal-derived ingredients, which leads to an inconsistency of messaging across the markets. Labels with terms that are deemed misleading or confusing, such as “cholesterol-free eggs,” may not only lead to intervention from regulators, but also confound consumers. It is vital to support compliance with established regulatory requirements with indicia for transparency and consumer education. A proposal to facilitate consumer education in the market could include descriptors that are standardized, such as “egg alternative”, as well as front-of-pack disclosures of nutrition.

## 7. Conclusions and Future Directions

This review demonstrates that egg alternative development has evolved from single-function substitution into multifunctional formulation strategies. Individual proteins, hydrocolloids, starches, lipids, and processing treatments can mimic specific egg functionalities such as gelation, foaming, emulsification, binding, and water retention; however, no single ingredient can reproducibly duplicate the integrated functionality of a whole egg. Therefore, the current evidence supports the importance of the strategic design of protein–hydrocolloid combinations and appropriate processing technologies rather than single ingredient replacement.

From an application perspective, different egg products require different formulation priorities. For scrambled eggs and omelets, legume proteins and emerging proteins such as RuBisCO, mung bean, and cowpea, combined with thermogelling hydrocolloids where necessary, are promising strategies for developing heat-set protein networks; however, simultaneous matching of hardness, cohesiveness, water retention, and thermal behavior remains insufficiently demonstrated. For mayonnaise and other yolk-like emulsions, plant proteins combined with hydrocolloids, phospholipid-containing ingredients, or other interfacial stabilizers provide promising routes for controlling emulsification and rheology, but quantitative equivalence in emulsifying activity and stability, droplet-size distribution, viscosity, oxidative stability, and sensory mouthfeel remains to be established. For bakery applications, protein–polysaccharide and hydrocolloid systems can provide combinations of aeration, binding, moisture retention, and structural development, although simultaneous reproduction of batter rheology, gas-cell stabilization, product volume, crumb structure, and texture remains challenging. For meringue and other foam-dominated applications, protein-based foaming systems are promising, but foam overrun, drainage stability, thermal resistance, and final texture require further optimization. These application-specific differences demonstrate that the suitability of an egg alternative should be evaluated against the functional requirements of the intended product rather than by a single measure of overall “egg-like” performance.

There remain a number of measurable gaps across these systems. Future studies should report the performance of the alternatives against conventional egg controls under standardized or clearly defined conditions and quantitative endpoints, such as foam overrun and stability, emulsifying activity and stability, droplet size, viscosity and flow behavior, thermal transitions, gel strength, water-holding capacity, instrumental texture and sensory attributes. Nutritional equivalence also needs to take into account protein quality and nutrient bioavailability, such as that of vitamin B12, choline and vitamin D, especially where the replacement formulations significantly alter the composition of the original egg product. Moreover, economic feasibility, scalability, ingredient availability and environmental performance should be assessed at the level of the process and product-system and not only at the level of the ingredient.

Future research should therefore focus on (i) complementary protein combinations with enhanced functionality and nutritional quality; (ii) controlled protein–polysaccharide–lipid interactions; (iii) processing technologies such as high-moisture extrusion, precision fermentation and cold plasma with clear functional advantages; (iv) standardized quantitative benchmarks against conventional egg; and (v) integrated assessments of functionality, nutrition, sensory acceptance, cost and environmental performance. Moving away from qualitative claims of “egg-like” functionality, quantitatively validated design criteria for specific applications will be required to develop egg-alternative systems that are fit for purpose both technically and nutritionally.

## Figures and Tables

**Table 2:** Legume-based proteins used as egg alternatives.

Protein Sources	Main Objectives	Food Products	Functionality	Reference
Cowpea	Effects of drying on cowpea’s albumin (as an alternative to egg white)	Meringue cookies	↑ Foaming by freeze-dried albumin compared to spray-dried and vacuum-dried albumin ↑ Foam stability by freeze-dried albumin compared to spray-dried and vacuum-dried albumin	[89]
Soy protein isolates Mung protein isolates Chickpea protein isolates	Assessing the potential of mixtures of plant protein isolates as egg alternatives	Whole egg gel	↑ Rheological similarity to eggs by increasing soy protein isolate concentration	[91]
Dry-fractionated protein concentrates from chickpea Dry-fractionated protein concentrates from red lentil Dry-fractionated protein concentrates from mung bean	Assessing the use of dry-fractionated protein concentrates as egg alternatives in food formulations	Sponge cakes	↑ Similarity of specific volume and thickness to those of egg by increasing dry-fractionated protein contents ↑ Similarity of crumb structure to that achieved by egg when increasing dry-fractionated protein content ↑ sensory properties of products formulated with chickpea and lentil dry-fractionated protein-based cakes compared to mung bean-based cakes	[92]
Soy protein isolates Pea protein isolates Lupin protein isolates	Use of protein isolates as egg yolk alternatives in hollandaise sauce	Sauce	↑ Water binding of soy protein isolate compared to pea and lupin protein isolates ↑ Emulsification by heating compared to non-heated proteins ↑ Foam capacity of lupin compared to other proteins	[93]

The upward (↑) arrows represent “high/increase”.

**Table 5:** Currently commercialized plant-based egg products.

Product Name (Country of Production)	Formulation Details/Format	Key Features/Use Cases	Website Link
JUST Egg (USA)	Based on mung bean protein Liquid, pre-cooked folded patties	Suitable for breakfast dishes, sandwiches, cookies, and scrambled egg-formulations Mimics scrambled eggs No cholesterol	www.ju.st
Zero Egg (USA & Israel)	Based on soy, pea, chickpea, and potato proteins Available in form of liquid, patties, and scrambled	Meant to perform like egg in recipes: baking, scrambling, etc. Offers versatility; designed to work in multiple culinary applications No cholesterol	www.zeroegg.com
Crackd (United Kingdom)	Based on pea protein Liquid form	For diverse purposes Free from 14 major allergens Zero cholesterol Non-GMO ingredients	www.crackd.com
OnlyEg (Singapore)	Egg yolk-like feature Ready-to-eat poached whole egg	Designed to mimic styles like “sunny side up”, “soft boiled”, etc., i.e. more than just scramble or bind. Closer to appearance and presentation of classic eggs Good for those missing whole egg dishes Pre-shredded omelets Patties Tamagoyaki	www.onlyeg.com
Tamalga (France)	Microalgae-based formulation Powder form Whole egg substitute	Designed specifically for industrial/bakery/pastry use; replicates binding, emulsifying, gelling etc. Powder form is good for shelf stability Seems to perform well in many baking applications Reduces environmental footprint	www.algamafoods.com
Beleaf (USA)	Based on soy protein	For restaurants/cafes wanting the look & experience of a fried egg without animal products Visually compelling; fits breakfast menus Allergen-safe option for some diners Long shelf-life when frozen “Sunny side up” style alternative, frozen, targeted at foodservice	www.beleafvegan.com

## Data Availability

No new data were created or analyzed in this study. Data sharing is not applicable to this article.
